# A guide to ERK dynamics, part 1: mechanisms and models

**DOI:** 10.1042/BCJ20230276

**Published:** 2023-12-01

**Authors:** Abhineet Ram, Devan Murphy, Nicholaus DeCuzzi, Madhura Patankar, Jason Hu, Michael Pargett, John G. Albeck

**Affiliations:** Department of Molecular and Cellular Biology, University of California, Davis, U.S.A.

**Keywords:** biological networks, computational models, epidermal growth factor receptor, extracellular signal-regulated kinases, mitogen-activated protein kinases, receptor tyrosine kinases

## Abstract

Extracellular signal-regulated kinase (ERK) has long been studied as a key driver of both essential cellular processes and disease. A persistent question has been how this single pathway is able to direct multiple cell behaviors, including growth, proliferation, and death. Modern biosensor studies have revealed that the temporal pattern of ERK activity is highly variable and heterogeneous, and critically, that these dynamic differences modulate cell fate. This two-part review discusses the current understanding of dynamic activity in the ERK pathway, how it regulates cellular decisions, and how these cell fates lead to tissue regulation and pathology. In part 1, we cover the optogenetic and live-cell imaging technologies that first revealed the dynamic nature of ERK, as well as current challenges in biosensor data analysis. We also discuss advances in mathematical models for the mechanisms of ERK dynamics, including receptor-level regulation, negative feedback, cooperativity, and paracrine signaling. While hurdles still remain, it is clear that higher temporal and spatial resolution provide mechanistic insights into pathway circuitry. Exciting new algorithms and advanced computational tools enable quantitative measurements of single-cell ERK activation, which in turn inform better models of pathway behavior. However, the fact that current models still cannot fully recapitulate the diversity of ERK responses calls for a deeper understanding of network structure and signal transduction in general.

## Introduction

**Figure 1.**
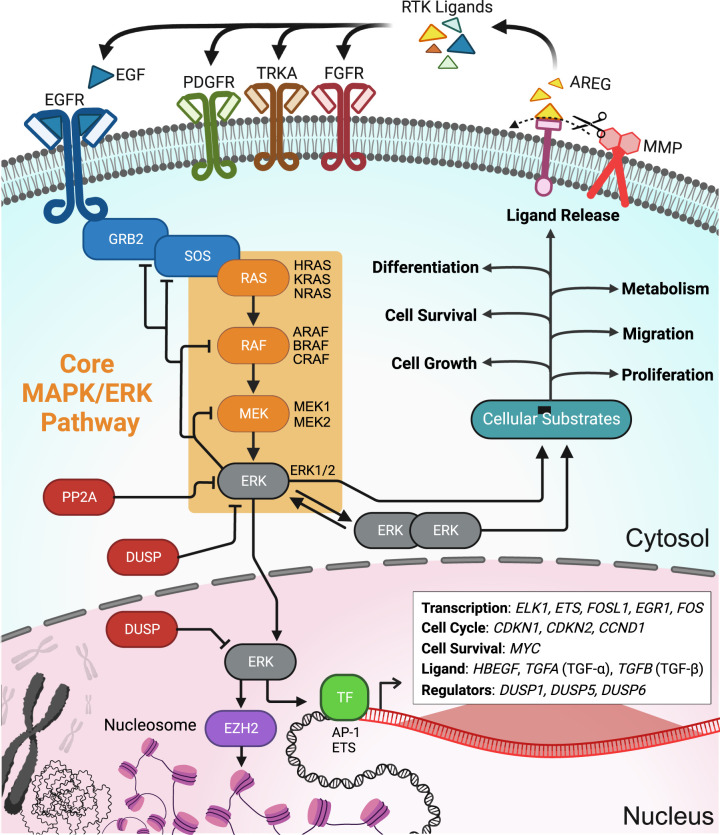
The central ERK signaling pathway. Initiation of the MAPK/ERK pathway begins with ligand binding of tyrosine receptor kinases (RTKs). This begins the phosphorylation cascade and activation of the core MAPK/ERK pathway consisting of RAS, RAF, MEK, and ERK (orange box, individual isoforms are listed). Active ERK can translocate to the nucleus, where it stimulates gene expression, or dimerize and phosphorylate cytoplasmic substrates. Depending on ERK dynamics, several gene expression programs can be activated, including cell cycle, cell survival, and ligand production (pathways bolded and specific genes listed in the box within the nucleus). Outside the nucleus, ERK regulates cytoplasmic proteins involved in cell growth, metabolism, and differentiation. Pathway termination is regulated by numerous phosphatases (PP2A and DUSPs), as well as several negative feedback loops mediated by ERK phosphorylation. For a comprehensive discussion of additional molecular details see [1]. TF, transcription factors.

The extracellular signal-regulated kinase (ERK) pathway (Figure 1) plays a widespread role in the development and physiology of animals [1]. ERK is a member of the mitogen-activated protein kinase (MAPK) family, which is found in all eukaryotes. Among the MAPK family, ERK1 (MAPK3) and ERK2 (MAPK1) have received a disproportionate amount of attention, owing to their overlapping and essential involvement in many processes that impact human health. Other ERK paralogs, including ERK3, ERK4, ERK5, and other MAPK family members including the JNK and p38 kinases, also play significant roles in these processes. Nonetheless, we focus here on developments in understanding the regulation of ERK1/2 activity, which is required for the proliferation of cancer cells, the formation of memory by neurons, and morphological changes in development, among many other examples. For more than two decades, it has been recognized that the frequency, duration, and amplitude of ERK activation are important in determining its effect on the cell [2]. Under some circumstances, ERK activation is more dynamic than that of JNK and p38 [3]. However, other studies have observed pulsatile JNK and p38 activities in response to cell stresses [4–7], suggesting this form of behavior is a common theme for MAPK pathways. Collectively, these dynamic behaviors appear to arise from the regulatory topology of the respective MAPK cascades, which contain numerous feedback loops [4,8].

Several early studies laid the conceptual groundwork for understanding the importance of ERK dynamics. In the 1990s, observations from several groups first established a relationship between ligand stimulation, the timing and duration of ERK activity, and cell fate [9]. Manipulating ERK activation patterns by different growth factors, receptor expression levels, or oncogenic mutants led to alternate cell fates [10]. In parallel, Ferrell et al. [11] showed that MAPK activation occurs in a highly switch-like manner in individual *Xenopus* oocytes. These results demonstrated that a pathway's output does not necessarily operate as a simple linear response to stimuli, but instead is shaped heavily by feedback, especially when viewed at the single-cell level [12]. Finally, it was found that the regulatory structure for a number of ERK target genes can make them sensitive to the duration of ERK activity [13–15]. Together, these concepts form the overarching framework for dynamics-based information encoding and decoding by the ERK pathway. In this review, we focus on the unique dynamic behavior observed for ERK and examine how it arises from the biochemical organization of the pathway. In a companion review, we look further into the impact of ERK dynamics on downstream processes and cell phenotypes.

Mathematical models have played an essential role in the study of ERK, providing a way to test questions that are not accessible experimentally and to explore possible mechanisms for dynamic behavior. In general, the flux of protein–protein interactions and modifications in the pathway can be represented as a system of ordinary differential equations (ODEs), which simulate pathway dynamics under different conditions. Historically, Ferrell and colleagues used such models to understand how MAPK pathways could exhibit the observed non-linear responses without explicit cooperativity and positive feedback [12]. This behavior is termed zero-order ultrasensitivity and occurs in MAPK systems when both the kinase and competing phosphatase molecules available are limited enough to become saturated [16]. Subsequently, the question of how transient ERK behavior arises under constant stimuli led to an expansion of MAPK models. Early evidence implicated the internalization of the epidermal growth factor receptor (EGFR) [17], but it was also argued that the transient assembly of signaling complexes at the EGFR could explain the observed transient kinetics [18]. Multiple models then explored the possibility of oscillations in activity due to feedback phosphorylation [19,20]. Orton et al. [21] elegantly summarized the early mathematical models of MAPK signaling, and the field of MAPK modeling continues to evolve, exploring the complex effects of feedback and more subtle concepts such as buffering of ERK by its substrates [22]. The concepts of transient, oscillatory, and excitatory behavior remain actively studied, especially with regard to distinguishing between true oscillations and pulsatile responses excited by fluctuating external stimulus. Throughout this review, we discuss the relevant mathematical models that can be used to understand the dynamic operation of the ERK pathway.

## Forms of dynamic ERK activity

Experimentally observed ERK dynamics can be grouped into several major categories (Box 1), including sustained, transient, peak with sustain, oscillatory, sporadic, and complex. These categories are not always clearly distinct, but they provide a useful framework for discussing ERK activity over time. In early studies, the PC-12 rat pheochromocytoma cell line served as a useful model system, as it responds with ligand-specific ERK dynamics: sustained activity to NGF stimulation and transient activity to EGF [23]. Importantly, these dynamics have phenotypic consequences resulting in cell proliferation and differentiation, respectively. At the time, population-level assays, such as immunoblots, were only able to provide rough estimates of ERK patterns, such as sustained activation lasting several hours, or transient activation peaking at ∼20 min before returning to baseline [9,10,24,25]. More complex ERK dynamics such as oscillations were postulated [19] but only became clearly observable with the development of fluorescent ERK biosensors [26,27]. These reporters are briefly summarized in the following section and have been reviewed in depth elsewhere [28].

Using live-cell reporters, Pertz and colleagues re-examined the classic PC-12 system, confirming the original findings from Marshall et al. but also uncovering substantial cell-to-cell variation [29]. This variation is extremely broad; Ryu et al. found both sustained and transiently responding cells at different proportions within any population of PC-12 cells, regardless of EGF or NGF stimulation. Further intricacies were revealed in the form of oscillations [30] and sporadic pulses [31,32] in growth factor-stimulated cells. The cell-to-cell variation also found in these systems made it clear why these diverse ERK activity forms were not measurable in immunoblot studies; because they occur asynchronously between cells, they are blurred in the average of thousands of cells in an immunoblot sample. In addition to distinguishing single-cell variation, live-cell assays also provide much greater time resolution, allowing dynamics to be closely tracked on the scale of minutes for many hours or even days, in contrast with the small number of time points typically captured in an immunoblot.

BOX 1. **Field Guide to Dynamic ERK Signaling**

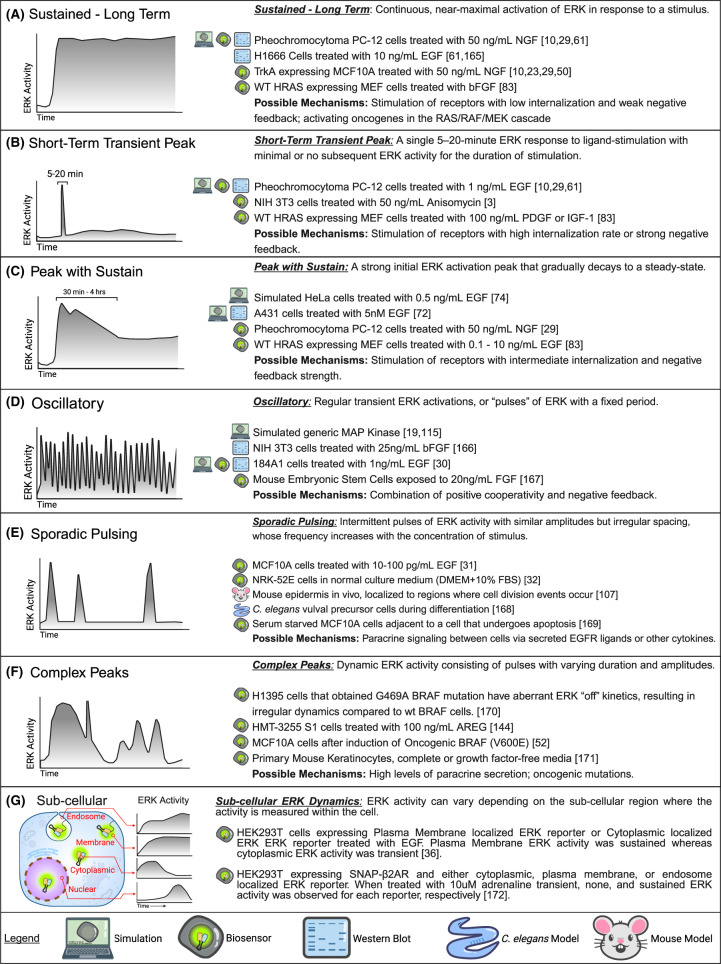

The subcellular distribution of active ERK within a cell is also an important facet of ERK dynamics. ERK sequestration to different subcellular regions can be a mechanism to regulate interactions between ERK and its substrates, altering the subset of targets that are phosphorylated [33,34]. For example, ERK translocation from the cytoplasm to the nucleus appears to be required for the phosphorylation of some substrates, such as the transcription factor ELK1 and subsequent induction of gene expression [35]. ERK biosensors localized to the plasma membrane and endosomes have begun to uncover examples of distinct subcellular ERK activity patterns. Within a particular cell, activity at the plasma membrane can be sustained, in contrast with the transient activation observed in the cytosol and nucleus [36]. However, complexities in the subcellular milieu remain yet to be fully resolved. ERK translocation is not necessarily required for the phosphorylation of ERK substrates within the nucleus [37,38]. It is possible for ERK to interact with and phosphorylate its substrates irrespective of their bulk localization because both ERK and its substrates such as ELK1 or FOS can shuttle between nucleus and cytosol on the scale of minutes [39–43]. Thus, even with the biosensors now available and the elegant work already performed, it must be recognized that interactions over space and time create many complex possibilities for the ERK signaling system [8]. Further work is still needed to resolve the full temporal and subcellular features of ERK activity dynamics.

## Advances in measuring ERK activity and remaining challenges

ERK dynamics are most easily detected by fluorescent protein-based ERK activity reporters (i.e. biosensors) which have recently been reviewed in detail [28]. The main categories of reporter include FRET-based (EKAR series), translocation-based (ERK-KTR; ERK-FP fusions), and degradation-based (FIRE) (outlined in Table 1). While the FRET-based ERK sensor has undergone many generations of improvements, the ERK-KTR, ERK-FP and FIRE reporters remain essentially unchanged (Table 1). Furthermore, as each reporter type has advantages and disadvantages, the choice of reporter used is critical when studying live-cell ERK activity. For instance, FRET-based ERK reporters are spectrally limited to fluorescent proteins (FPs) capable of FRET, such as CFP/YFP. Alternatively, translocation-based reporters use only a single FP of any color, providing much more flexibility to combine with other reporters or fluorescent markers [3,44]. Additional markers to distinguish the nucleus from cytosol are still needed to quantify translocation reporters, and cells with complex three-dimensional or dynamic shapes can be a significant challenge to accurately quantify. Reporters also vary in the timescale of ERK activity changes they can detect, with FRET reporters showing the fastest responses, followed closely by translocation-based reporters, and degradation reporters being the slowest. While rapid reporter responses are needed to accurately distinguish closely grouped pulses of ERK activity, the slow responses of a degradation-based reporter can be very useful for measuring the integrated activity of ERK over time [31,45,46].

**Table 1:** Genetically Encoded ERK activity Biosensors grouped based on their sensing modality

Reporter Type	Reporter name (aliases)	Subcellular Resolution of ERK activity	Dynamic Range	Response Time (approx.)	Sensitivity	Fluorescent Protein(s)	Cell Shape Sensitivity	CDK Sensitivity	Reference
FP-fused ERK	FP-ERK (BFP-ERK, (GFP-ERK)	No	+	3min	+	BFP, GFP *	Yes	No	[52,79,185]
Degron	FIRE	No	+++	150 min	+++	mVenus *	No	No	[31]
Kinase Translocation Reporter (KTR)	ERK-KTR (ERKKTR, ERKTR)	No	++++	3 min	+++	Clover *	Yes	Yes	[3]
Forster Resonance Energy Transfer (FRET)	EKAR	Yes	+	1 min	++	mVenus, mCerulean	No	Yes	[27]
	EKAREV (EKARev, EKAR-EV)	Yes	++	1 min	++	YPet, ECFP	No	Yes	[181]
	EKAR2G1	Yes	+	1 min	+	cp173Venus, cp227mTFP1	No	Yes	[182]
	EKAR-TVV	Yes	++	1 min	++	cp173Venus-Venus, mTurquoise	No	Yes	[53,182]
	RAB- EKARev	Yes	++	5 min	NA	ddRFP-A, ddRFP-B	No	Unknown	[183]
	FPX-EKAR	Yes	+ (50% of EKARev)	5 min	+	Red-ddFP, Green-ddFP, ddFP (B)	No	Unknown	[184]
	EKAR3	Yes	+	1 min	++	YPet, mTurquoise2	No	Yes	[50]
	EKAR4	Yes	+++	1 min	++	ECFP, YPet	No	No	[36]
	EKAR-EN4 (EKAREN4)	Yes	+++	1 min	++	ECFP, YPet	No	No	[48]
	EKAR-EN5 (EKAREN5)	Yes	+++	1 min	+++	YPet, mTurquoise2	No	No	[48]

Fluorescent protein fused ERK (FP-ERK) translocates partially to the nucleus when phosphorylated by MEK allowing average cellular activity to be estimated by the nuclear to cytosolic fluorescence ratio. The Degron reporter (FIRE) is stabilized upon phosphorylation by ERK such that its fluorescence indicates ERK activity on a scale of 3-12 hours. Upon phosphorylation, KTR reporters shift in their preference for nuclear import vs. export allowing their nuclear vs. cytosolic ratio to reflect the average cellular ERK activity on the scale of minutes. FRET reporters shift in conformation upon phosphorylation by ERK allowing the ratio of fluorescent protein intensities to reflect subcellular ERK activity on the scale of minutes. Subcellular resolution of ERK activity: yes indicates the capability for the reporter to be localized to different subcellular compartments and to therefore reflect the activity of a specific region. Response Time: an estimate of time from ERK activation to apparent localization/fluorescence change. Cell Shape Sensitivity: the apparent read-out of translocation-based reporters can be shifted independently of ERK by changes in cell shape. Cyclin Dependent Kinase (CDK) sensitivity: Some reporters can be phosphorylated independently of ERK by Cyclin Dependent Kinases. * any fluorescent protein can be theoretically used.

In most cases, the specificity of ERK reporters is high, as judged by the ability of either MEK or ERK inhibitors to eliminate their signal. However, one notable exception is the tendency of FRET and translocation-based reporters to show a non-ERK-specific increase in activity late in the cell cycle. This non-specific response is attributable to the fact that the ERK substrate sequences used in many of the existing reporters can also be phosphorylated by cyclin-dependent kinases (CDKs) that are most active in the G2 and M phases [32], causing a slow increase in reporter signal that is resistant to MEK or ERK inhibitors and rapidly disappears following cell division [47]. In our experience, the onset of this non-specific activity varies between cell lines; some cells show an increase in non-specific signal 1–2 h prior to mitosis while others show a much longer period of accumulation. A recent set of FRET-based reporters derived from the EKAR-EV reporter, EKAR-EN4 and EKAR-EN5, addressed this problem by mutating two residues in the target phosphorylation sequence to eliminate the CDK affinity [48].

An ongoing challenge for accurate reporter readouts lies in quantifying the intensity of ERK activity. This is an inherently difficult problem, as ‘ERK activity' at any given time is not a uniform parameter across the cell. In addition to spatial variability, different endogenous substrates can be phosphorylated to different extents, depending on the affinity of the substrate-kinase interaction [49]. Thus, any individual reporter is inherently limited to a single ‘perspective' on ERK activity, while the set of endogenous ERK substrates represents multiple perspectives. Combining multiple ERK reporters in the same cell has been a useful exercise to show how the same pulse of ERK activity can be received differently by alternate targets [50–52]. These studies show that FRET and translocation-based ERK reporters agree in large part, but they also reveal subtle differences in on-rate and off-rate. Another key difference is in the measured amplitude of ERK activity. Dual readouts highlight systematic differences in dynamic range between reporters. For example, the FRET reporter EKAR3 shows greater sensitivity than ERK-KTR to small ERK activity changes but saturates easily [51]. While the dynamic range of FRET-based reporters has increased [48,53], a head-to-head comparison between the newest FRET reporters and translocation reporters to assess their relative advantages has not yet been performed. Altogether, these differences emphasize the caveat that the amplitude of ERK reporter signals must be interpreted with caution and not as an absolute linear measurement. We discuss these quantitative issues in more depth in Box 2.

BOX 2. **Rigor and Challenges in Quantification and Analysis**

Reporter Calibration For true quantitative measurements of ERK activity, two problems must be dealt with. First, the reporter signal itself must have its linear range of response characterized. This can be done by western blotting, to relate the fraction of the reporter in its phosphorylated form to its readout detected by FRET [83,158]. When performed carefully, reporter signals can be interpreted quantitatively, relative to the maximal signal, and any non-linear regions of the readout can be identified. Second, the reporter readout must be linked to the level of ERK activity in the cell. This calibration can be approached by relating ERK FRET readouts to immunoblots on parallel samples that measure the fraction of ERK phosphorylation or endogenous ERK substrate phosphorylation. However, a crucial caveat is that ERK reporters indicate not simply ERK activity, but instead the balance of ERK activity relative to any phosphatase activity on the reporter's ERK target site. The rapid reversibility of reporter signals upon ERK inhibition indicates high cellular phosphatase activity, and it seems reasonable that these phosphatases are the same ones that act on endogenous ERK substrates. However, this assumption has not been established experimentally. Any change in this phosphatase activity will affect the relationship between ERK activity and the observed reporter signal. This complicating factor can be approached by mathematically modeling both ERK and phosphatase effects on the reporter, or by empirically determining the relationship between phosphorylated ERK and the reporter signal [83]. While often overlooked, phosphatase activity may be one of the main drivers of heterogeneity in observed ERK readouts, both within and between cell types.

**Figure 2.**
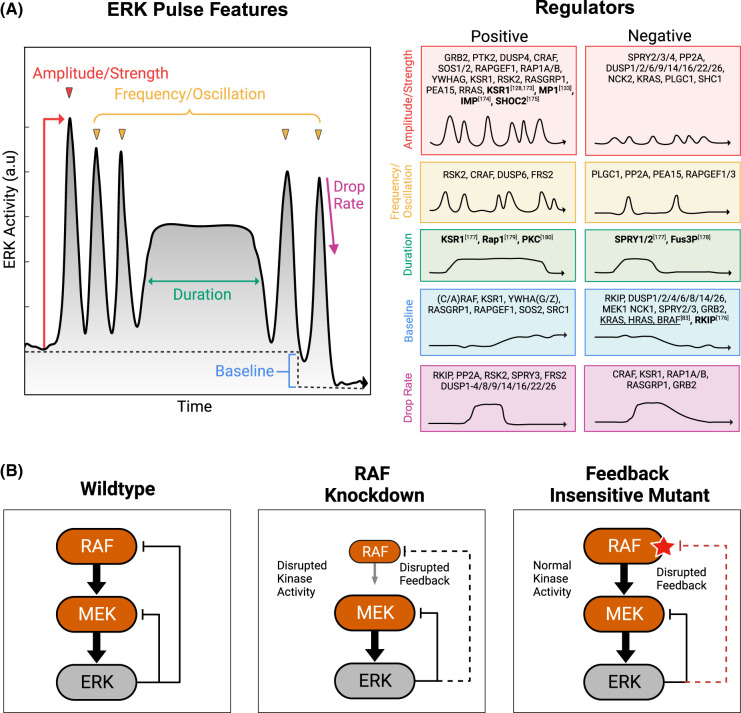
Feedback mutants and regulators of ERK pulse dynamics. (**A**) Dynamic features of ERK activity and genes that have been shown to positively or negatively regulate them. This list is curated from experiments where ERK activity features were measured after knockdown or knockout (KD/KO) of respective genes. KD/KO of positive regulators resulted in a net decrease or delay of ERK activity, while KD/KO of negative regulators resulted in a net increase or acceleration of ERK activity. Most experiments were performed at single-cell resolution [81], or from western blot experiments (indicated in bold) [2]. (**B**) Comparison of experimental techniques to investigate the strength of negative feedback. *Left*: ERK inhibits both MEK and RAF. *Middle*: Experimental knockdown of Raf weakens negative feedback from ERK; however, signaling from RAF to MEK will also be disrupted. *Right*: Feedback-insensitive mutants only weaken the negative feedback from ERK, and allow for wild-type RAF to MEK signaling.

Quantifying features in time series data Once live-cell data is collected, one must choose the appropriate technique to mathematically describe, or ‘featurize', the time-dependent signal of ERK activity. Several mathematical methods are available to extract information from time series data [159]. Pulse detection algorithms identify peaks of signal activity and then quantify parameters such as signal amplitude, pulse duration, or frequency (Figure 2A) [160,161]. Other methods include Fourier and wavelet transformation [162,163], which decompose time series measurements into simpler components (which, added together, reconstruct the original signal). With any of these methods, the challenge lies in identifying the information that is most relevant for the cellular process under study, whether it be the amplitude, duration, average, or another aspect of ERK activity. Typically, it is necessary to experiment with more than one method to quantify the relationship of interest.

Clustering cells by dynamics Parsing cells with similar reporter activity is often necessary as a first step during analysis. This task is not trivial as cellular kinetic data frequently have overlapping distributions, and thus determining the appropriate number of clusters is often arbitrary. A critical consideration is whether to predefine the number of clusters or allow the algorithm to determine the final number of groupings. There are many clustering functions to choose from, including K-means clustering, hierarchical clustering, K-nearest neighbor, and other deep learning-based methods. Another important consideration is which distance metric to use; dynamic time warping has proved to be one useful approach, which allows signals that are similar in shape but have different timing to be grouped together [164]. Each of these approaches require significant user input which must be guided by awareness of algorithm limitations and the structure of the data. As a result, clustering can be challenging to implement in exploratory research.

Deep learning and neural networks offer a more sophisticated approach to classify dynamic signaling behaviors. Rather than directly breaking down signals into unique characteristics, neural networks are trained to recognize distinguishing features in the data. A recent example of this is CODEX, which can recognize dynamic ‘prototypes' for signal behavior that can be used to group similarly behaving cells [157]. This method allows a computer to learn which patterns distinguish signal activity between specified categories, such as treatment conditions. Although these methods allow for analysis of large, multidimensional datasets, it can be difficult for humans to understand the abstract patterns that the algorithms learn. CODEX resolves this issue by providing prototypical time trajectories for each of the categories it identifies. An additional advantage is that CODEX can be used on datasets where multiple biosensors are measured in the same cell. Thus, with the increasing size and complexity of reporter datasets, deep learning methods provide an attractive tool to facilitate data interpretation.

Another current challenge lies in extracting meaningful information from the hundreds or thousands of cells that are interrogated in a typical live-cell imaging experiment. The first step in this process is the extraction of ERK activity ‘traces' from image datasets, which can now be performed automatically using various segmentation and tracking algorithms [54–56]. While this step was often rate-limiting in the past, advances in computational image analysis have made it routine. In particular, machine learning software such as StarDist and CellPose have greatly increased the reliability of automated cell recognition [57,58]. Tracking algorithms, such as uTrack [59] and EllipTrack [60], link cells from one image frame to the next, creating a time-series vector for each cell. Typically, it is possible to track over 90% of cells in each experiment; however, tracking efficiency is reduced by abnormal cell morphology, over-confluency, fast migration, or cell death. Despite these limitations, recent studies have used data from thousands or even hundreds of thousands of cells to draw statistically well-supported conclusions. Subsequent challenges emerge in the analysis of high-content time-series data, which we briefly discuss in Box 2.

## Modeling the mechanisms driving dynamics

The question of how different forms of ERK dynamics are generated at the molecular level has captured scientific interest for at least 30 years [9]. Approaches to this question have spanned structural analysis, subcellular localization, and mass-action kinetic modeling [33,61–66]. Many mechanistic details can shape the dynamic behavior of ERK, and here we group these mechanisms into several overarching concepts and discuss the evolution of mathematical models that explore these factors. Computational models play an increasingly essential role in this question because the complexity of multiple layers of regulation makes it difficult or impossible to predict system behavior from intuition alone. A major caveat that applies across these studies is that many mathematical models pre-date the ability to track ERK activity in live cells. Consequently, many published models, although intended to represent a prototypical cell, have been fit only to population-average data, which does not always accurately represent the true behavior of any individual cell. Thus, conclusions from models must be interpreted with caution in cases where it is unknown how single cells differ from the mean.

### Predominance of RTKs in setting ERK dynamics

From the earliest studies of ERK signaling, it was observed that ligands for different receptor tyrosine kinases (RTKs) can specify distinct activity kinetics [24]. These receptor-specific patterns can be attributed either to differential binding of adaptor and RAS-family G proteins to the receptor [67], or to differences in the kinetics of receptor dimerization, internalization, degradation, and recycling [9,68]. Dimerized EGFR molecules perform autophosphorylation of their partners, which targets them for internalization by both clathrin-mediated and clathrin-independent mechanisms [69,70]. Although the receptor may continue to signal from endosomal compartments of the cell, this internalization ultimately results in EGFR inactivation and transient ERK activation (Box 1B) [68,71]. Numerous mathematical models of ERK signaling have incorporated the mechanisms of receptor processing as a focus of regulation [21,61,72–78]. These models enabled the exploration of how receptor internalization rates determine the duration of ERK activity and predict responses to different EGF levels.

The importance of receptor kinetics is underscored by converging evidence that ERK activity tracks very closely with RTK activity. When ERK activity is stimulated by light-induced optogenetic constructs upstream of RAS, the activity follows the intensity of light stimulation with very little lag or adaptation [79,80]. This ‘memoryless' behavior is surprising given that several downstream negative feedback loops (detailed in the next section) are operative under these conditions and would be expected to complicate the signal dynamics. However, a strong correlation between upstream initiation and ERK output has been observed in multiple systems, regardless of whether the signaling is initiated at the level of RAS or the intracellular domain of RTKs [81]. Further corroborating this concept are data showing that ERK activity terminates within seconds to minutes upon RTK inhibition [50,73], and that ERK activity tracks dynamically with receptor phosphorylation across different receptors [82].

A further line of evidence for the importance of receptors in dynamics is that oncogenic or activating mutations in proteins downstream of the receptor, including RAS, RAF, or MEK, generally promote sustained ERK activity in single cells (Box 1A) [52,83]. In contrast, manipulating the activity of the receptors results in changes in pulse frequency. Together, these data argue that the tendency toward transient or sustained activity of ERK is primarily a reflection of the activation and deactivation of the ligand-bound receptor, in at least several commonly studied cell types. However, under more atypical experimental conditions, the regulation of EGFR internalization can result in surprising behavior. Under conditions in which EGF is slowly ramped to high concentrations, receptors become down-regulated and fail to activate ERK [84]. This adaptation persists for hours, and even withdrawal of EGF for several hours and subsequent re-stimulation does not elicit ERK activation. Thus, receptor-level regulation also acts as a noise filter to reduce spurious ERK activity in the face of incremental or gradual ligand changes. More generally, this study implies that an important area to refine models of EGFR internalization and feedback is in the response to complex but physiologically relevant stimulation patterns that deviate from simple bolus treatments.

It is also important to note that while studies of ERK activation and dynamics have focused heavily on receptor tyrosine kinase signaling, ERK can be activated in a number of other ways. G protein-coupled receptor (GPCR) signaling activates ERK through arrestin [85], a key regulatory scaffold protein that binds to GPCR tails upon activation, and through arrestin-independent mechanisms [86]. Ligands for different GPCRs can generate distinct ERK and Protein Kinase B (AKT) activity responses [87]. Protein kinase C is also capable of activating RAF [88], as are cellular oscillations of calcium [89], providing additional inputs to ERK signaling. Physiologically, it is likely that cells simultaneously receive multiple stimuli, and understanding the dynamics induced by these combinations at the single-cell level is an underexplored area for further study.

### Additional regulation by downstream negative feedback loops

Another essential feature of ERK regulation is an intricate negative feedback structure. Active ERK can negatively regulate several upstream targets, including EGFR [90], MEK1 [91], RAF [92], or SOS [93,94]. Still another level of negative feedback is the ERK-mediated transcriptional induction of phosphatase genes, such as the dual specificity phosphatases (DUSPs) and MAPK phosphatases (MKPs) [95]. Increased expression of DUSPs and MKPs leads to dephosphorylation of the MAP kinases, reducing their activity. The net result of these seemingly redundant negative feedback mechanisms is a strong tendency of ERK activity to fall sharply within 15–30 min after its peak activation, even independently of the receptor internalization described above, to enforce the transient pulse shape observed in many cell types (Box 1B). In contrast, systems with weaker collective negative feedback show sustained signaling (Box 1A) [20,61,67]. Studies combining both modeling and experiments have built a consensus that negative feedback loops vary in their relative importance, explaining the diverse ERK dynamics found across different cell types [72,96,97].

One of the most thorough efforts to deconvolve feedback mechanisms in ERK dynamics was a pathway-wide RNAi screen of 50 MAPK genes by Dessauges et al. [81]. With its large-scale and detailed analysis employing optogenetic stimulation at different points in the pathway, this landmark study provided two important conclusions. First, a number of subtle changes in ERK dynamics resulted from knocking down certain genes, including CRAF, RSK2, PP2A, and PLCG1 (Figure 2A), several of which are involved in negative feedback. Some of these knockdowns led to increased oscillatory behavior, while others moderately increased ERK amplitude. Second, this study underscores the remaining challenge of disentangling highly redundant signaling systems. In many cases, ERK activation was not affected by the knockdown of core pathway genes such as ERK2, GRB2, or SOS2, likely because additional isoforms of these proteins maintained their function. Perhaps most strikingly, the authors found that even this extensive dataset was still insufficient to fully specify a multi-feedback computational model. Thus, the redundancy of negative feedback loops continues to be a formidable challenge for both experiments and modeling.

While computational models can capture basic ERK kinetics using one or more of these feedback loops [19,72,76,98], it is difficult to verify that these models capture the underlying biology. Due to the redundancy of feedback circuitry (Figure 2B, left), isolating single feedback loops is experimentally difficult. Simple knockdown or overexpression experiments are often limited in their ability to test feedback loop functions because they would change both the forward and the feedback effects of the protein within the loop (Figure 2B, middle). Ideally, feedback nodes could be isolated experimentally by replacing the proteins involved with feedback-insensitive versions (Figure 2B, right). Such isolation would require either editing multiple sequences in endogenous genes or expressing a mutated protein while simultaneously knocking out the endogenous protein, both of which would be highly time-consuming. The closest examples to date ablate specific feedback loops via phospho-insensitive RAF [92,99,100] or SOS mutations [93,101]. Consequently, current computational models likely suffer from overfitting due to the large number of loops in the system and limited experimental data to constrain these parameters. Future experiments aimed at accurately disentangling individual feedback nodes, without altering the protein's forward signaling activity, will refine models and improve prediction performance.

In addition to simply terminating pathway activation, negative feedback plays an important role in producing linear ERK responses that are robust to noise [64,102]. Because ERK inhibits upstream pathway components, the system takes on the topology of a negative feedback amplifier, a design frequently used in engineering to stabilize system output and reduce sensitivity to environmental perturbations. Acting in this fashion, pathway inputs that would normally saturate ERK output instead show a graded linear response over a wide range of stimuli [64,102]. Finally, another function of negative feedback is that it can render the amount of ERK activity output insensitive to the total ERK protein level [103]. Together, these studies highlight the importance of negative feedback in setting the system-level input–output properties of ERK activity and the need for models to represent the multiple feedback loops accurately. A simplified interpretation that reconciles many of the existing observations is that negative feedback loops within the RAF–MEK–ERK cascade act on the scale of seconds or minutes and provide linearity and robustness to the input–output behavior of this module, while feedback at the receptor level varies the input to the cascade on a longer time scale, creating the overall form of the dynamics. However, this concept has yet to be fully tested, both computationally and experimentally.

### Pulsatile and oscillatory behavior due to cooperativity

In many systems, the ERK cascade exhibits evidence of cooperativity - that is, a steeply non-linear response curve to ligands that tends toward full activation once stimulated [12,104,105]. In experiments using single-cell assays, ERK activity often transitions rapidly from fully off to maximally active, with few intermediate responses observed [12,32]. Cooperativity is important in allowing the ERK pathway to act as an excitable system in which activity can propagate spatially, either within a cell or from cell to cell. This form of activity is referred to as a trigger wave, and has been observed in various types of monolayer cultures, both *in vitro* and *in vivo* [106–109]. In the slime mold *Dictyostelium*, the RAS-linked signaling network displays excitability that allows regions of RAS activity to propagate within individual cells [110].

The most comprehensive study of cooperative MAPK behavior has been carried out in *Xenopus* oocytes, where cooperative activation is driven by positive feedback from MAPK to the MAPKKK Mos [11,111]. However, in other systems, the source of cooperativity has been more difficult to identify definitively. It has been suggested that the requirement for dual phosphorylation of MEK and ERK enables cooperative behavior of the cascade, and modeling of these effects shows that they are sufficient to create switch-like behavior or oscillations in ERK/MAPK activity (e.g. Box 1D) [63]. Another potentially important positive feedback occurs at the level of SOS, a guanine nucleotide exchange factor that mediates RAS activation by RTKs [112,113]. SOS has two binding sites for RAS — one at which it catalyzes guanine nucleotide exchange on RAS, and one at which GTP-bound RAS binds and allosterically enhances exchange activity at the first site. This allostery creates a positive feedback loop, which has been proposed as the source of cooperative ERK activation in mammalian cells [104]. However, the observations that optogenetic stimulation either at the receptor level or the SOS level fail to elicit cooperative activation of ERK suggest that these mechanisms alone are insufficient for cooperativity [81]. Thus, similar to the situation of redundant negative feedbacks, there remains substantial difficulty in unambiguously establishing contributions of individual positive feedback mechanisms in most cell types examined to date.

Despite the ambiguity in the molecular mechanism, it is likely that some combination of negative feedback and cooperativity underlies the oscillatory or highly pulsatile behavior that has been observed for ERK in various systems [30,114]. The first demonstration of such a possibility used a model in which high cooperativity (also known as ultrasensitivity) was coupled to negative feedback from ERK to RAF to produce oscillatory behavior [19]. A number of other models have confirmed that such combinations can produce oscillatory behavior. In a more recent example, Kochańczyk et al. [115] constructed a MAPK pathway model with one positive feedback from Ras to SOS, and three negative feedbacks from ERK acting on MEK, RAF, and SOS. They found that the positive feedback from Ras to SOS allows for bistable pathway activation, and the negative feedback from ERK to SOS then refashions the network's bistable behavior into oscillatory patterns of ERK activation. In this model, negative feedback from ERK to MEK and RAF primarily modulates the shape of ERK activity pulses. Finally, similar models are supported by additional work from Arkun and Yesemi [116], who argue that bistability and switch-like behavior arise from positive feedback from Ras to SOS, but add that internal negative feedback from phosphatases allows for dampened oscillations.

### Signal amplification and regulation through scaffold proteins

MAPK activity dynamics can also be shaped by the assembly of signaling complexes via scaffold proteins, which simultaneously interact with multiple MAPK pathway components. Scaffolds can control both the spatial distribution and activation of ERK within the cell and the temporal characteristics of activity by facilitating kinase-substrate interactions or shielding kinases from dephosphorylation [117–119]. Scaffolds in the MAPK pathway were initially found to be essential for *S. cerevisiae* pheromone responses, where Ste5 was identified as a tether for multiple MAP kinases and later recognized for its role in shaping both graded and switch-like signaling [120–122]. Multicellular organisms lack obvious homologs of Ste5, but have other genes that may play similar functions. KSR1 homologs were first identified in *Drosophila and C. elegans* as positive regulators of the MAPK pathway [123,124]. KSR1 is a pseudokinase with homology to RAF whose catalytic activity has been debated; however, it was found to be capable of forming a complex with CRAF, MEK, and ERK [125–127]. Deletion of KSR1 in mouse embryonic fibroblasts reduces the intensity and duration of ERK activation [128]. In single cells, Dessauges et al. [81] demonstrate that KSR1 positively regulates ERK activity's amplitude, baseline, and adaptation (drop rate) but not its oscillations (Figure 2A). Another protein, SHOC2, promotes the interaction of RAS and RAF, and enhances the intensity of ERK activation [129]. Further complicating our understanding, experimental and computational evidence suggest that overexpression of some scaffolds can actually hinder MAPK activation, suggesting that optimal concentrations of scaffolds are required for efficient signaling [119,132]. Therefore, while KSR1, SHOC2, and other potential scaffolds such as IQGAP and MP-1 contribute to the strength and duration of ERK signaling [132,133], their significance relative to other regulators in generating the differences in ERK dynamics between cell types or between individual cells remains largely unexplored. Additional modeling analysis of pathway-wide datasets [81] could be used to address this gap.

### Autocrine/paracrine signaling as a source of sporadic pulses

While feedback and cooperativity can explain regular oscillations in ERK activity, irregular patterns of pulses (Box 1E,F) indicate a strong source of variability. Several lines of evidence suggest that autocrine and paracrine signaling through EGFR plays a dominant role in driving irregular pulsatile dynamics. Epithelial cells secrete numerous EGFR ligands [134], each eliciting distinct ERK activities. For example, high-affinity ligands, such as transforming growth factor α (TGF-α) rarely escape capture by the secreting cell's own receptors, and thus act primarily as an autocrine signal [135]. Lower-affinity ligands such as Amphiregulin (AREG) can diffuse more broadly to stimulate surrounding cells. The release of these ligands is controlled by matrix metalloproteinases (MMPs) on the cell surface that cleave the membrane anchor motif to release the soluble mature forms into the extracellular space [136]. MMPs are in turn stimulated by ERK activity, which effectively forms a positive feedback loop that operates across intracellular and extracellular compartments [32,137]. In addition to canonical EGFR ligands, other growth factors, including those from the fibroblast growth factor (FGF) family or G-protein coupled receptor (GPCR) ligands stimulate ERK and act in a paracrine manner [87,138,139]. The combination of these different ligands and the irregular timing of their release create a dynamically evolving microenvironment for the neighboring cells.

An additional layer of complexity arises from the fact that different EGFR ligands can trigger distinct patterns of ERK activity even though they signal through the same receptor. Freed et al. [66] examined ligand-specific EGFR dimer interactions and found that high-affinity ligands such as EGF or TGF-α create highly stable EGFR dimers, whereas low-affinity ligands such as Epiregulin and Epigen (EREG and EPGN) form weakly bound asymmetric dimers. The varying stability of these complexes results in differences in internalization rate, effectively altering the strength of a key negative feedback. Strong EGFR binders (e.g. heparin-binding EGF-like growth factor, betacellulin) target all EGFRs for lysosomal degradation and attenuate the signal [140]. As a result, EGFR molecules bound to EREG and EPGN are less subject to internalization and drive more sustained ERK signaling [140]. Furthermore, differences in ligand dissociation from internalized EGFR allows the receptors to be recycled to the plasma membrane surface rather than broken down, permitting rapid re-activation by ligand and the potential for sustained ERK activation [140,141]. This multitude of activation mechanisms further diversifies the ERK responses that result from paracrine stimulation.

In *in vivo* imaging studies, some form of dynamic ERK pulses, resembling those described in cell culture, have been observed in every case where single-cell resolution was available. The patterns of pulses vary depending on the tissue and organism. Examples of focal points of ERK activity that radially spread to neighboring cells include the mouse epidermis [107] and *Drosophila* embryonic epithelium [142]. In some cases, ERK activity only travels limited distances (3–4 cell diameters), suggesting that propagation is limited by diffusion of the ligand. However, in regenerating fish scales, wound healing, or cultured MDCK epithelial cells, waves of ERK activation travel much farther, spreading out across dozens of cell layers. In these cases, ERK activity causes shedding of EGFR ligands via MMPs, allowing for continued propagation of the wave [106–108,143]. Other cell systems show rapid, sporadic patterns of well-defined pulses with limited spatial correlation, suggesting multiple overlapping sources [31,32]. At the extreme end of this continuum, cells containing oncogenic mutations show a complex and seemingly stochastic pattern of ERK activity without clearly separated pulses, which has been linked to increased secretion of AREG, a paracrine EGFR ligand [48,52,144]. In nearly all of these cases, EGFR inhibition eliminates ERK pulses, confirming the importance of receptor-level regulation of these patterns and dynamics. Thus, paracrine ligand secretion underlies a variety of highly dynamic ERK behavior.

Several mathematical models have been developed to simulate the propagation of ERK activation between cells [32]. For instance, a spring model was used to investigate ERK-driven collective cell migration. In this model, ERK activity increases the length of each cell and subsequently changes cell density and decreases myosin light chain (MLC) phosphorylation. The model indicates that as ERK waves propagate through cells, MLC dephosphorylation is sufficient for collective cell migration in the opposite direction of the ERK wave, whereas cell density is not sufficient [143]. This model was restricted to observations in a one-dimensional monolayer. Therefore, the spring model was transformed into a continuum model, which allows for a two-dimensional analysis that accurately represents the 2D epithelial cell movement. The continuum model averages the heterogenous and noisy properties of individual cells in order to successfully recapitulate tissue-level dynamics driven by single cells [145]. Finally, biophysical models further our understanding of how monolayer mechanics coupled to ERK translate to polarity changes and active cell migration [146].

### Cell states create variability in ERK responses

Another prominent feature of ERK activation revealed by biosensors is cell-to-cell variation in activity patterns. Even in cases where genetically identical cells respond to controlled spatial differences in stimulating ligands, there is substantial divergence in the timing and intensity of ERK activation. Studies investigating this phenomenon have found that the variation can be accounted for by pre-existing differences in cell state, also termed ‘extrinsic noise', rather than true stochastic behavior of the pathway, or ‘intrinsic noise' [147]. This finding is consistent with results from several other signaling pathways [148] and confirmed by a recent study measuring dozens of cell state parameters, including local cell density, cell shape, and expression of various non-pathway markers [149]. The latter study demonstrated that cell state, as indicated by factors such as calreticulin, Sec13, and cell density may exert an even larger effect on a given cell's ERK activation (as well as for many other signaling pathways) as compared with different concentrations of EGF. This concept helps to explain a disparate set of findings that ERK pathway activation depends strongly on actin cytoskeletal protrusions [150], the presence of caveolin pits in the plasma membrane [151], and the rate of glycolysis [152]. If all of these ‘non-canonical' mechanisms each impact ERK activation, the pathway can be considered not only as an output of growth factor stimulation, but also as an integrated index of both intracellular and extracellular factors.

## Conclusion

The diversity of ERK dynamics helps to explain how this ubiquitous pathway plays a variety of cell-specific roles in controlling cell proliferation, differentiation, and migration. Collectively, the work highlighted here demonstrates that ERK activation dynamics are well positioned to provide acute sensing of the extracellular microenvironment, allowing cells to respond in unique ways to paracrine signals, cell density, and the extracellular matrix. When connected to pathway outputs, such as gene expression, that are selectively responsive to different dynamic patterns, the ERK pathway makes it possible for the cell to continuously adjust its state and behavior based on its physical context. In the companion review, we consider the ‘output' side of this function, exploring how dynamics regulate gene expression. We also examine the potential for pharmacological inhibitors of the ERK pathway to promote different cellular functions depending on how they affect ERK dynamics.

Fully understanding and exploiting the ERK signaling ‘code' will depend on accurate quantitative models. The rich history of pathway models that we discuss here has provided an excellent start in capturing the main mechanisms driving dynamic ERK activity. Nonetheless, as the most recent work shows, a complete model that accurately predicts the effects of pharmacological and genetic perturbations remains some distance away [81]. While existing models provide the conceptual building blocks to understand how dynamic behaviors arise, many cell systems contain several of these mechanisms operating together. As noted above, predictive models of highly redundant systems are challenging to validate, especially when relatively few experiments precisely dissect of the component mechanisms. Furthermore, even in the absence of mutations, genetically identical cells can diverge in their dynamics due to variations in the copy numbers of pathway proteins [153]. Such variation can explain the observed differences between cell types in an organism, and the heterogeneity of cells within the same tissue. Fully modeling these differences would require information on the hundreds of parameters (i.e. protein concentrations) that vary between contexts, which remains experimentally challenging.

The new technologies highlighted here, including improvements in biosensors, image processing, and large dataset analysis, will likely be critical in overcoming the remaining obstacles. Machine learning is an exploding field that has rapidly expanded into biology. From predicting protein structure, cell segmentation, and improving CRISPR guide RNA design, neural networks have pushed the boundaries of many fields [154–156]. Recently, convolutional neural networks have been used to identify ERK patterns and characterize signaling motifs in single cells [81,157]. These newer models are able to recognize objective and abstract patterns in large-scale data; therefore, they are an approach that may fully connect signaling, gene expression, and cell fates. Future work should be aimed at creating a model that connects network topology and the functional and phenotypic consequences of signal propagation. Specifically, how do the positive regulators of the pathway shape the spatial and temporal activation and deactivation of ERK? What features of the pathway are most important for regulation, and which are redundant? Furthermore, how important is the pathway topology for generating dynamic patterns of gene expression? Although it is unlikely there will be one universal model that represents all aspects of the pathway, future computational models can likely succeed in capturing the majority of the signaling network circuitry and simulating the full range of dynamic behaviors of ERK.

## Open Access

Open access for this article was enabled by the participation of University of California in an all-inclusive *Read & Publish* agreement with Portland Press and the Biochemical Society under a transformative agreement with UC.

## Abbreviations

A431: epidermoid carcinoma cell line
AREG: amphiregulin
BRAF: v-Raf murine sarcoma viral oncogene homolog B1
CRAF: RAF proto-oncogene serine/threonine-protein kinase
CRISPR: clustered regularly interspaced short palindromic repeats
DMEM: Dulbecco's Modified Eagle Medium
DUSPs: dual specificity phosphatases
EGF: epidermal growth factor
EGFR: epidermal growth factor receptor
EKAR: extracellular signal-regulated kinase activity reporter
EPGN: Epigen
EREG: Epiregulin
ERK: extracellular signal-regulated kinase
ERK2: mitogen-activated protein kinase coded by MAPK1 gene
ERK-FP: extracellular signal-regulated kinase-fusion protein
ERK-KTR: extracellular signal-regulated kinase translocation reporter
FBS: fetal bovine serum
FGF: fibroblast growth factor
FIRE: fluorescent InsP3-responsive element
FRET: fluorescence resonance energy transfer
Fus3P: the mitogen-activated protein kinases promoting G1 arrest (*S. Cervevisiae*)
G proteins: guanine nucleotide-binding proteins
GPCR: G-protein coupled receptors
GRB2: growth factor receptor bound protein 2
GTP: guanosine triphosphate
H1395: human lung adenocarcinoma epithelial cell line
H1666: human bronchoalveolar carcinoma epithelial cell
HEK293T: human kidney epithelial cell line
HeLa: human cervical cell line from Henrietta lacks
HMT-3255 S1: human breast epithelial cells
HRAS: Harvey rat sarcoma viral oncogene homolog
IGF-1: insulin-like growth factor 1
IMP: insulin-like growth factor 2 messenger RNA-binding proteins
KSR: kinase suppressor of Ras
MAPK: mitogen-activated protein kinase
MAPKKK: mitogen activates protein kinase kinase kinase
MCF10A: human breast epithelial cell line
MDCK: Madin-Derby canine kidney
MEF: mouse embryonic fibroblasts
MEK: mitogen-activated protein kinase kinase
MKPs: MAPK phosphatases
MLC: myosin light chain
MMPs: matrix metalloproteinases
MP-1: MEK Parter 1
NCK2: noncatalytic region of tyrosine kinase, Beta
NGF: nerve growth factor
NIH3T3: fibroblast cell line
NRK-52E: rat kidney epithelial cell lines
ODE: ordinary differential equation
PC-12: rat pheochromocytoma cells
PEA-15: phosphoprotein enriched in astrocytes
PKC: creatine phosphokinase
PLCG1: phospholipase C, Gamma 1
PP2A: protein phosphatase 2A
PTK2: protein tyrosine kinase 2
RAF: rapid accelerated fibrosarcoma
Rap1: Ras-proximate-1
RAPGEF1: Rap guanine nucleotide exchange factor 1
RAS: rat sarcoma virus
RASGPR1: RAS guanyl-releasing protein 1
RKIP: Raf kinase inhibitory protein
RNA: ribonucleic acid
RNAi: RNA interference
RRAS: Ras-related protein R-Ras
RSK2: ribosomal protein S6 kinase 2
RTK: receptor tyrosine kinases
Shc1: SHC adaptor protein 1
SHOC2: Soc-2 suppressor of clear homolog
SNAP-β2AR: self-labeling protein tag-β2-adrenergic receptor
SOS: son of sevenless
SPRY: Sprouty
TGFα: transforming growth factor alpha
TrkA: tropomyosin receptor kinase A
YWHAG: 14-3-3 protein gamma protein
YWHAZ: tyrosine 3 monooxygenase activation protein zeta
